# Intracortical inhibition is modulated by phase of prosthetic rehabilitation in transtibial amputees

**DOI:** 10.3389/fnhum.2015.00276

**Published:** 2015-05-19

**Authors:** Brenton Hordacre, Lynley V. Bradnam, Christopher Barr, Benjamin L. Patritti, Maria Crotty

**Affiliations:** ^1^Department of Rehabilitation, Aged and Extended Care, Repatriation General Hospital, Flinders UniversityAdelaide, SA, Australia; ^2^Applied Brain Research Laboratory, Centre for Neuroscience, School of Medicine, Flinders UniversityAdelaide, SA, Australia; ^3^Discipline of Physiotherapy, School of Health Sciences, Flinders UniversityAdelaide, SA, Australia; ^4^Discipline of Physiotherapy, Graduate School of Health, University of TechnologySydney, NSW, Australia

**Keywords:** transcranial magnetic stimulation, amputation, transtibial, motor cortex, human, paired-pulse, gait, rehabilitation

## Abstract

Reorganization of primary motor cortex (M1) is well-described in long-term lower limb amputees. In contrast cortical reorganization during the rehabilitation period after amputation is poorly understood. Thirteen transtibial amputees and 13 gender matched control participants of similar age were recruited. Transcranial magnetic stimulation was used to assess corticomotor and intracortical excitability of M1 bilaterally. Neurophysiological assessments were conducted at admission, prosthetic casting, first walk and discharge. Gait variability at discharge was assessed as a functional measure. Compared to controls, amputees had reduced short-latency intracortical inhibition (SICI) for the ipsilateral M1 at admission (*p* = 0.01). Analysis across rehabilitation revealed SICI was reduced for the contralateral M1 at first walk compared to discharge (*p* = 0.003). For the ipsilateral M1 both short and long-latency intracortical inhibition were reduced at admission (*p* < 0.05) and prosthetic casting (*p* < 0.02). Analysis of the neurophysiology and gait function revealed several interesting relationships. For the contralateral M1, reduced inhibition at admission (*p* = 0.04) and first walk (*p* = 0.05) was associated with better gait function. For the ipsilateral M1, reduced inhibition at discharge (*p* = 0.05) was associated with poor gait function. This study characterized intracortical excitability in rehabilitating amputees. A dichotomous relationship between reduced intracortical inhibition for each M1 and gait function was observed at different times. Intracortical inhibition may be an appropriate cortical biomarker of gait function in lower limb amputees during rehabilitation, but requires further investigation. Understanding M1 intracortical excitability of amputees undertaking prosthetic rehabilitation provides insight into brain reorganization in the sub-acute post-amputation period and may guide future studies seeking to improve rehabilitation outcomes.

## Introduction

Transtibial amputation of a lower limb has a significant effect on human function and requires extensive rehabilitation to restore mobility using a prosthetic limb. Despite lengthy rehabilitation (Hordacre et al., 2013b), many amputees are unable to achieve high levels of activity and mobility in the community and remain at high risk of falling (Miller et al., 2001; Hordacre et al., 2014a). While poor rehabilitation outcomes may be related to factors such as advanced age and comorbidities, there is recent evidence that the pattern of reorganization of the primary motor cortex (M1) may be related to functional outcomes (Hordacre and Bradnam, 2013; Hordacre et al., 2014b). Investigating cortical reorganization immediately after amputation and determining the relationship between neurophysiology and function will facilitate appropriate selection of brain-targeted interventions that may improve rehabilitation outcomes.

Reorganization of the contralateral cortex following amputation was first investigated in animal models. In the non-human primate, both contralateral primary sensory and motor cortices reorganize topographically to occupy cortical territories formerly representing the amputated limb or digit (Rasmusson and Turnbull, 1983; Merzenich et al., 1984; Donoghue et al., 1990). Expansion of cortical representations and increased corticospinal excitability have also been observed in human amputees using functional magnetic resonance imaging (MRI) or transcranial magnetic stimulation (TMS; Cohen et al., 1991; Fuhr et al., 1992; Chen et al., 1998a; Röricht et al., 1999; Simões et al., 2012). Greater corticospinal excitability is most likely associated with reorganization at the cortical level, with evidence suggesting spinal excitability is unchanged (Fuhr et al., 1992; Chen et al., 1998a). Paired-pulse TMS studies indicate reorganization of the contralateral cortex in long-term amputees is mediated by modulation of gamma-aminobutyric acid (GABA) receptor activity (Chen et al., 1998a; Schwenkreis et al., 2000). GABA is an important inhibitory neurotransmitter crucial to the maintenance of cortical motor representations (Jacobs and Donoghue, 1991). Although not yet demonstrated in lower limb amputees, an increase in *N*-methyl-D-aspartate (NMDA) receptor activity in upper-limb amputees has been observed (Schwenkreis et al., 2000). NMDA plays an important role in regulating the excitability of interneuronal circuits (Ziemann et al., 1998; Schwenkreis et al., 1999) and cortical reorganization is dependent on NMDA receptor-mediated activity (Kano et al., 1991; Garraghty and Muja, 1996). Therefore, both GABA and NMDA-receptor mediated responses may underpin cortical reorganization in post-acute lower limb amputees.

The ipsilateral cortex also reorganizes following amputation. Human TMS studies indicate increased ipsilateral corticospinal excitability in long-term amputees (Hordacre et al., 2014b). Similar to the contralateral cortex, modulation of GABAergic inhibition has been observed in the ipsilateral M1 in post-acute and long-term lower limb amputees (Capaday et al., 2000; Hordacre and Bradnam, 2013). In an amputee case study undertaken during the post-acute phase, reduced GABAergic inhibition was associated with greater function assessed with the amputee mobility predictor (Hordacre and Bradnam, 2013). There is a suggestion that bilateral cortical reorganization may be driven by activity in transcallosal projections from the reorganizing cortex contralateral to the amputated limb (Kew et al., 1994; Werhahn et al., 2002), however, modulation of transcallosal inhibition has not been observed in the amputee brain (Hordacre et al., 2014b).

Amputee neurophysiological investigations have predominantly focussed on long-term amputees to date, and little is known about how cortical reorganization evolves during the rehabilitation period. Recent evidence suggests a close relationship between function and cortical neurophysiology in lower limb amputees (Hordacre and Bradnam, 2013; Hordacre et al., 2014b). A better understanding of longitudinal modulation of corticomotor excitability and intracortical inhibition and facilitation shortly after amputation may assist rehabilitation practice. Therefore, the purpose of this study was to investigate longitudinal bilateral reorganization of M1 in transtibial amputees during the period of prosthetic rehabilitation from admission to discharge. We explored the relationship between neurophysiological measures using TMS at key time points of rehabilitation and gait function at discharge using spatial-temporal gait variability measures (Vanicek et al., 2009; Parker et al., 2013). It was hypothesized lower limb amputees would demonstrate bilateral reorganization of M1 mediated by GABAergic inhibition, with reduced GABAergic inhibition associated with better gait function at discharge.

## Materials and Methods

### Participants

Thirteen unilateral transtibial amputees [10 male, mean age 61.1 (range 45–85) years] admitted for prosthetic rehabilitation at one regional rehabilitation hospital in Adelaide, SA, Australia (Hordacre et al., 2013a) were recruited. Participants were screened for clinical characteristics including stump-length and phantom pain, which was assessed with the pain component of the Prosthetic Evaluation Questionnaire (Boone and Coleman, 2006). Those with phantom pain were excluded as neuroactive drugs are administered at the recruiting hospital to control phantom pain. These drugs are known to alter assessment of cortical excitability (Rizzo et al., 2001). A comparator group of 13 gender matched control participants of similar age were purposively recruited. Limb dominance of control participants was assessed with the Edinburgh Handedness Inventory which included two questions regarding lower limb dominance (Oldfield, 1971), and the non-dominant limb was modeled as the amputated limb. Potential participants with contraindications for TMS, including metallic implants, a history of seizures and medications known to alter central nervous system excitability were excluded (Rossi et al., 2009). Ethical approval was provided by the Southern Adelaide Clinical Human Research ethics committee and all participants provided written informed consent in accordance with the Declaration of Helsinki.

### Protocol

To assess changes in brain neurophysiology transtibial amputee participants attended one afternoon laboratory session at each of four pre-selected key time points during their rehabilitation. Assessments were conducted at time of admission to rehabilitation, prosthetic casting, first walk, and discharge from rehabilitation, individualized for each amputee (Hordacre and Bradnam, 2013; Hordacre et al., 2013a). During TMS, participants were seated comfortably with hip and knee joints flexed to 90°. A seated knee-extension task was used to unilaterally pre-activate the rectus femoris (RF) muscle prior to each TMS pulse. In the latter stages of rehabilitation when amputees were provided with a prosthesis, TMS tests were conducted with the prosthesis removed for consistency. Muscle activation during the knee extension task was monitored at 10–15% maximal voluntary contraction with visual feedback of raw electromyography (EMG) signal from the RF. TMS pulses were triggered during muscle contractions using Signal software (Signal v5.09, Cambridge Electronic Design, Cambridge, UK) at a frequency of 0.2 ± 10% Hz.

### Electromyography

Surface EMG was recorded from each RF using 10 mm-diameter Ag/AgCl electrodes (Ambu, Ballerup, Denmark). Electrodes were placed 2 cm apart over the muscle bellies, with the distal electrode positioned ∼12 cm superior to the patella. A 20 mm-diameter reference Ag/AgCl electrode (3M Health Care, Saint Paul, MN, USA) was placed over the patella. Prior to affixing the electrodes the skin was prepared for optimal contact by shaving to remove hair, lightly abrading and cleaning with alcohol. EMG signals were sampled at 2000 Hz (CED 1401; Cambridge Electronic Design, Cambridge, UK), amplified (CED 1902; Cambridge Electronic Design, Cambridge, UK), band-pass filtered (20–1000 Hz) and stored for oﬄine analysis (Signal v5.09, Cambridge Electronic Design, Cambridge, UK).

### Transcranial Magnetic Stimulation

Single monophasic TMS pulses were delivered using a single Magstim 200 stimulator, and paired monophasic TMS pulses were delivered using two stimulators connected to a BiStim^2^ unit (Magstim Company, Dyfed, UK). Both single and paired-pulse TMS were delivered using a flat 70 mm wing diameter, figure eight coil. The coil was held tangentially over the scalp with the handle pointing 30° posterior-medially in the transverse plane positioned over the contralateral cortex 1 cm posterior and 1.5 cm lateral to the vertex (Hordacre and Bradnam, 2013). The ‘hotspot’ for evoking maximal responses in the contralateral active RF was then determined separately for the primary motor cortex (M1) contralateral to the amputated side (M1CON) and M1 ipsilateral to the amputated side (M1IPSI) by systematically moving the coil over a 1 cm grid from this location and marked on the scalp. Active motor threshold (AMT) was determined separately for each M1 as the minimum stimulus intensity eliciting a 100 μV MEP in five of ten stimuli in the contralateral RF (Rossini et al., 1994). For single-pulse TMS, 16 MEPs were evoked at 120% AMT. MEP amplitude was used to calculate a laterality index (LI) to assess the balance of corticomotor excitability of descending projections to the amputated (control non-dominant) and non-amputated (control dominant) limbs. Positive LI values indicate relative greater excitability of contralateral projections to alpha-motoneurons innervating RF of the amputated (control non-dominant) limb. Negative LI values indicate relative greater excitability of contralateral projections to alpha-motoneurons innervating RF of the non-amputated limb (control dominant limb). The equation to calculate LI was;

$$LI=\frac{(MEP\,\;amplitude\,\;\;M1CON\,-MEP\,\;amplitude\,\;\;M1IPSI)\;\;\;\;\;\;\;\;}{(MEP\,\;amplitude\,\;M1CON+MEP\,\;amplitude\,\;\;M1IPSI)\;\;\;\;\;\;\;\;}$$

Three paired-pulse TMS measures of intracortical excitability were assessed, short latency intracortical inhibition (SICI), long latency intracortical inhibition (LICI), and intracortical facilitation (ICF). For each measure, 16 non-conditioned and 16 conditioned MEPs were evoked in randomized order. The test stimulus was set to produce a half maximum MEP (50% MEPmax), ensuring the test MEP was evoked from the linear portion of the stimulus response curve for each individual (Devanne et al., 1997). This method allows for MEP facilitation or suppression during paired-pulse TMS while avoiding ceiling or floor effects. SICI was assessed using three conditioning stimulus intensities (70, 80, and 90% AMT), with an inter-stimulus-interval of 2 ms, generating a SICI recruitment-curve (Peurala et al., 2008; Talelli et al., 2011). LICI was assessed using a suprathreshold conditioning stimulus (50% MEPmax) delivered 100 ms before the test stimulus (McDonnell et al., 2006). ICF was assessed using a conditioning stimulus intensity of 80% AMT and two inter-stimulus-intervals of 10 and 15 ms (Talelli et al., 2011).

For both single and paired-pulse TMS, MEPs where pre-stimulus root mean square EMG (rmsEMG) were two standard deviation (SD) above or below the mean were removed prior to averaging to ensure consistency of MEP responses (number of responses removed ranged between 0 and 3). From the retained traces MEPs were measured peak-to-peak and conditioned MEPs were normalized to non-conditioned MEPs to determine a ratio of facilitation or inhibition.

### Spatial-Temporal Gait Variability

Spatial-temporal gait variability was assessed at discharge from rehabilitation as a measure of gait function. Previous studies demonstrate that increased levels of gait variability are associated with falls histories in transtibial amputees (Vanicek et al., 2009; Parker et al., 2013; Hordacre et al., 2014c) and it was therefore considered an appropriate indicator of poor functional outcome. Gait was assessed using an instrumented GAITRite walkway (CIR-Systems Inc., Sparta, NJ, USA). Embedded pressure sensors captured individual footfall data over an active area of 4.9 m × 0.6 m. Participants completed 10 consecutive passes over the GAITRite at their self-selected comfortable walking speed. Data were collected at 120 Hz and analyzed using GAITRite software (version 4.5). The coefficient of variation (CV), calculated as SD divided by the mean, was used to assess variability of spatial-temporal parameters. Gait variability measures may be affected by walking speed, therefore individual gait trials were normalized for walking speed prior to calculation of CV (Hordacre et al., 2014c). Step-time, step-length and step-width variability of the amputated limb were reported as these measures are sensitive markers of gait function in transtibial amputees (Hordacre et al., 2014c).

### Data Analysis

The normality of data was checked with a Shapiro–Wilk normality test. ICF assessed in M1CON and M1IPSI were log transformed to achieve normality. Significance level was set at *p* ≤ 0.05 and SPSS software was used for all statistical analyses (IBM corp. Released 2010. IBM SPSS Statistics for Windows, Version 19.0, Armonk, NY, USA).

### Demographics and Clinical Characteristics

Age was compared between amputees and controls with an independent *t*-test. Descriptive statistics were used to report stump-length and time since amputation for the key rehabilitation milestones.

### Association of Cortical Reorganization with Prosthetic Rehabilitation

The optimal conditioning stimulus intensity responsible for producing maximal SICI for each participant was determined at admission and used in off-line analysis of SICI for comparison across rehabilitation time points. This optimal conditioning stimulus determined at admission was kept constant throughout rehabilitation. Similarly, the optimal inter-stimulus-interval responsible for producing maximal ICF for each participant was determined at admission and analyzed across rehabilitation time points. This optimal inter-stimulus-interval determined at admission was kept constant throughout rehabilitation. For SICI, LICI, and ICF in each M1, conditioned and non-conditioned MEPs were compared at admission to rehabilitation with paired *t*-tests to confirm presence of inhibition (SICI and LICI) and facilitation (ICF). SICI, LICI, ICF for each M1 and the LI were separately compared between controls and amputees at admission and discharge with independent *t*-tests. For amputees, AMT, SICI, LICI, ICF for each M1 and LI were individually assessed with a 4 SESSION repeated measures ANOVA (rmANOVA). *Post hoc* tests explored significant effects and were corrected for multiple comparisons using a modified Bonferroni test (Rom, 1990). The corrected *p*-values for successive *post hoc* analyses were 0.05, 0.025, 0.0169, and 0.0127. For amputees, pre-trigger rmsEMG was compared between sessions with a 2 CONDITIONED × 4 SESSION rmANOVA for SICI, LICI, ICF and a 4 SESSION rmANOVA for MEP amplitude (used to calculate LI) for each hemisphere. For controls, pre-trigger rmsEMG for conditioned and non-conditioned MEPs for SICI, LICI, ICF were individually compared with paired *t*-tests for each hemisphere. For amputees, paired-pulse test MEP amplitude was compared between sessions with a 4 SESSION rmANOVA for SICI, LICI, ICF for each hemisphere.

### Association of Cortical Reorganization with Gait Function

The association between discharge gait function (normalized step-time, step-length, and step-width variability) and neurophysiology measures across rehabilitation time points (LI, SICI, LICI, ICF) were assessed for each test session with linear regression analyses. Linear regression models were controlled for age and stump-length as these factors can affect gait function (Gonzalez et al., 1974; Kang and Dingwell, 2008; Callisaya et al., 2010). Independent variables were analyzed using ‘Enter’ method in SPSS. These regression models were exploratory in nature and given the small sample sizes, analyses were not corrected for multiple comparisons so not to miss reporting potentially important results worthy of further exploration (Perneger, 1998; Nakagawa, 2004).

## Results

### Demographics and Clinical Characteristics

There was no difference in age between amputees (61.1, SD 12.4 years) and controls (58.9, SD 9.8 years; *p* = 0.49). Mean stump-length of amputees was 18.1 (SD 3.4) cm. Amputees were admitted to rehabilitation a median 14 (range 7–18) days following amputation surgery. Median time to prosthetic casting was 32 (range 14–41) days, to first walk was 50 (range 32–75) days and to discharge from rehabilitation was 87 (range 47–99) days. There were no interruptions to the rehabilitation program or transfers back to acute care for illness for any amputees during the study.

### Association of Cortical Reorganization with Prosthetic Rehabilitation

For amputees, the optimal conditioning stimulus intensities for evoking SICI in M1CON were 70% AMT (*n* = 7), 80% AMT (*n* = 2) and 90% AMT (*n* = 4); and for M1IPSI were 70% AMT (*n* = 3), 80% AMT (*n* = 4) and 90% AMT (*n* = 6). The optimal ISI for evoking ICF in M1CON were 10 ms (*n* = 4) and 15 ms (*n* = 9); and for M1IPSI were 10 ms (*n* = 7) and 15 ms (*n* = 6). For control subjects, the optimal conditioning stimulus intensities for evoking SICI in M1CON were 70% AMT (*n* = 4), 80% AMT (*n* = 4) and 90% AMT (*n* = 5); and for M1IPSI were 70% AMT (*n* = 7), 80% AMT (*n* = 1) and 90% AMT (*n* = 5). The optimal ISI for evoking ICF in M1CON were 10 ms (*n* = 5) and 15 ms (*n* = 8); and for M1IPSI were 10 ms (*n* = 8) and 15 ms (*n* = 5). At admission to rehabilitation analysis of M1CON found the conditioned MEP was significantly smaller than the non-conditioned MEP for SICI [*t*_(12)_ = 2.10, *p* = 0.04] and LICI [*t*_(12)_ = 2.41, *p* = 0.03], and larger for ICF [*t*_(12)_ = 1.85, *p* = 0.05]. For M1IPSI, the conditioned MEP was significantly smaller than the non-conditioned MEP for LICI [*t*_(12)_ = 1.92, *p* = 0.05]. However, for M1IPSI there was no significant difference between conditioned and non-conditioned MEPs for both SICI [*t*_(12)_ = 0.72, *p* = 0.25] and ICF [*t*_(12)_ = 1.10, *p* = 0.15] indicating modulation of intracortical excitability in this hemisphere early following amputation.

There was reduced SICI in M1IPSI in amputees at admission (mean 1.03, SD 0.2) compared to controls [mean 0.89, SD 0.1; *t*_(24)_ = 2.64, *p* = 0.01]. There was no difference in the other neurophysiological measures between amputees at admission and controls (all *p* ≥ 0.11). There was no difference between amputees at discharge and controls for any neurophysiological measure (all *p* ≥ 0.15).

**FIGURE 1:**
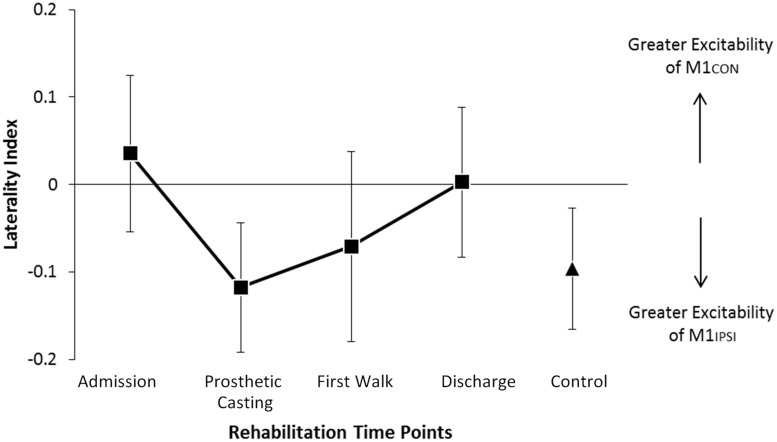
**Modulation of LI across rehabilitation time points**. There was no significant difference in LI across rehabilitation or compared to control participants. In amputees negative LI indicates greater excitability of M1 driving the non-amputated limb. Negative LI in controls indicates greater excitability of M1 driving the dominant limb. Cortical excitability in amputees was balanced at discharge. Error bars represent standard error.

**FIGURE 2:**
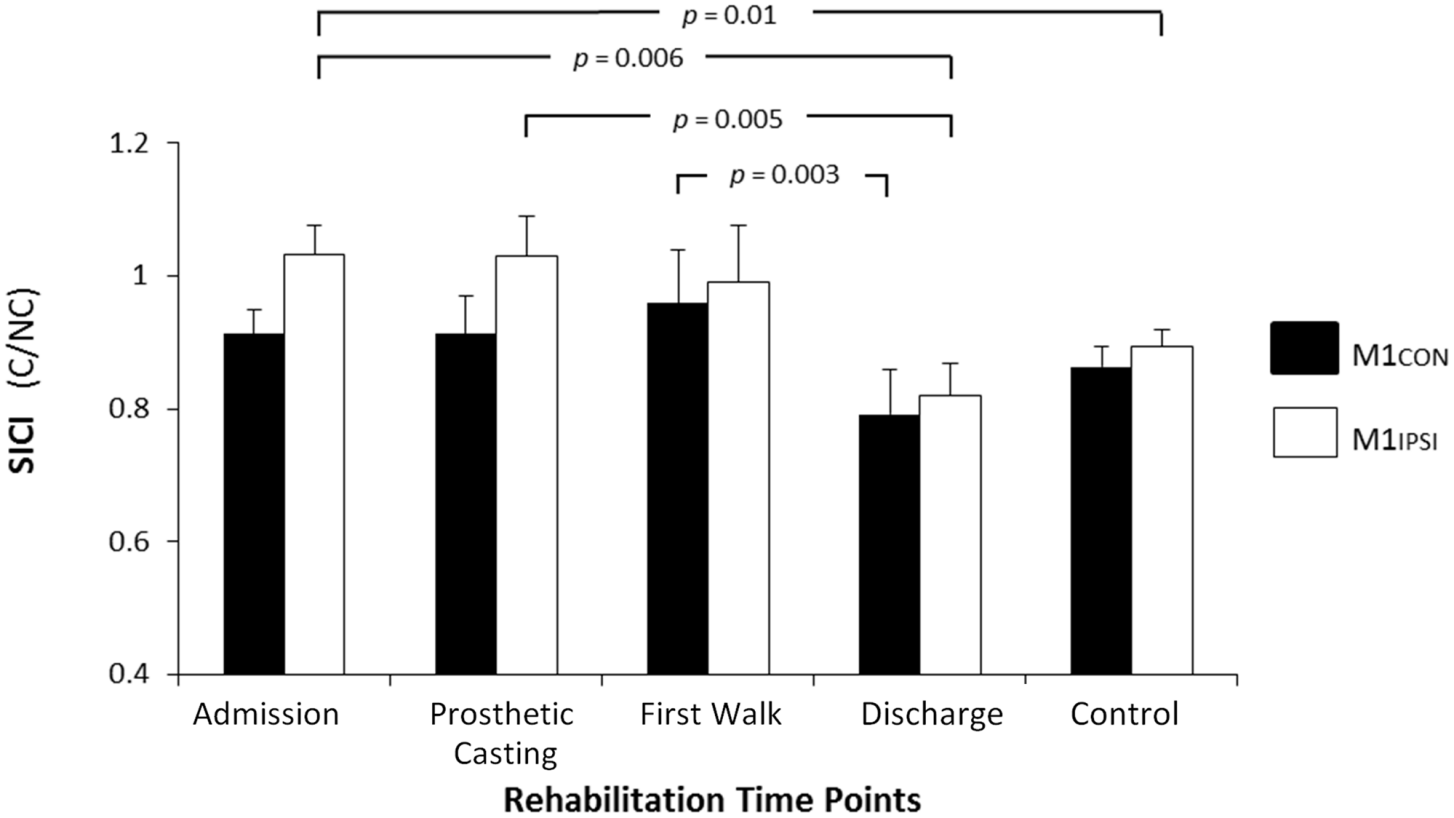
**Modulation of SICI across rehabilitation time points for M1CON and M1IPSI**. For M1CON, there was a reduction in SICI at first walk compared to discharge. For M1IPSI, there was a reduction in SICI at admission and prosthetic casting compared to discharge. SICI was also reduced at admission compared to the control participants. There were no differences between amputees and controls at discharge. Error bars represent standard error. C, conditioned motor evoked potentials; NC, non-conditioned motor evoked potentials.

**FIGURE 3:**
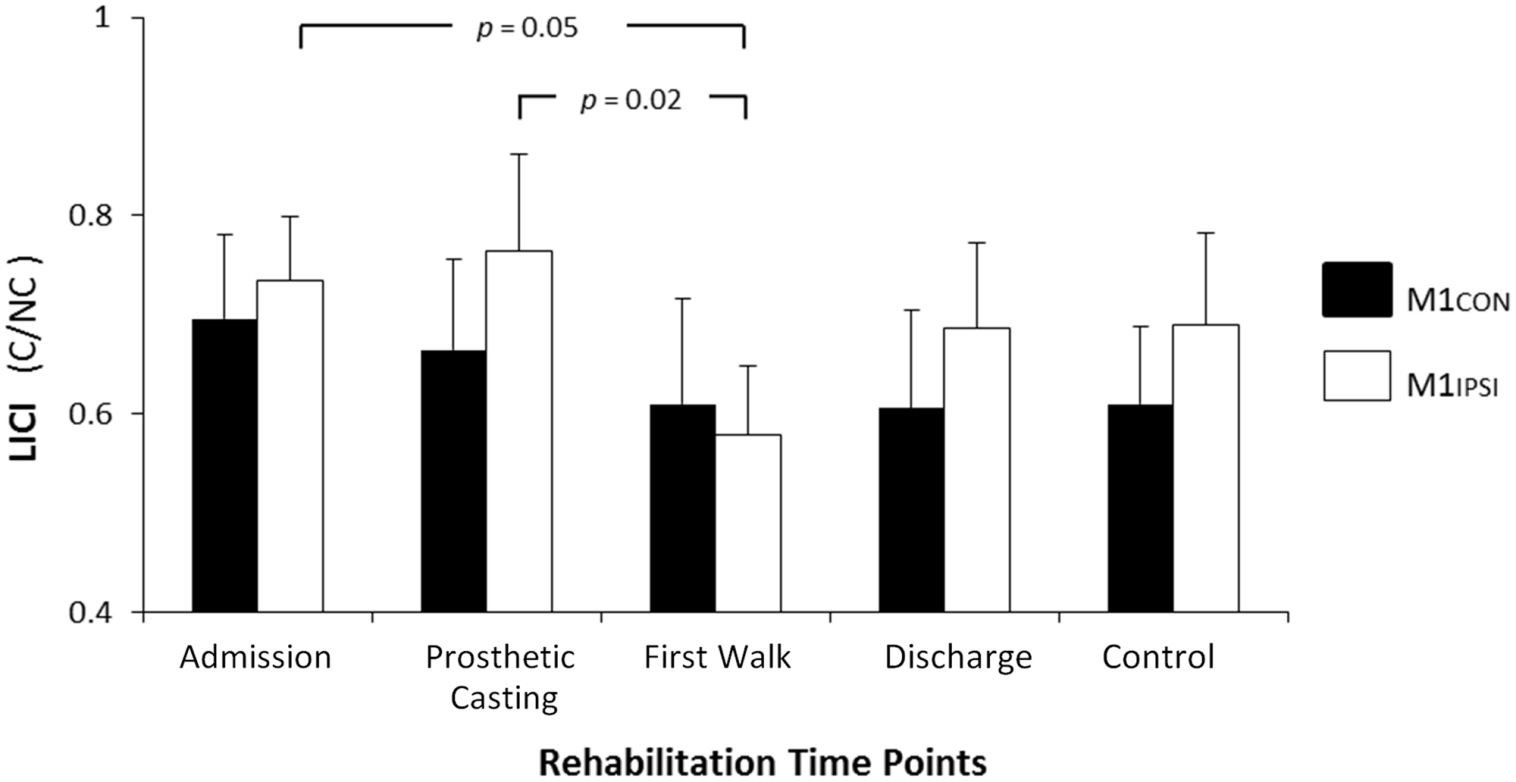
**Modulation of LICI across rehabilitation time points for M1CON and M1IPSI**. There was no significant modulation of LICI across rehabilitation for M1CON. For M1IPSI, there was a reduction in LICI at admission and prosthetic casting compared to first walk. Error bars represent standard error. C, conditioned motor evoked potentials; NC, non-conditioned motor evoked potentials.

**FIGURE 4:**
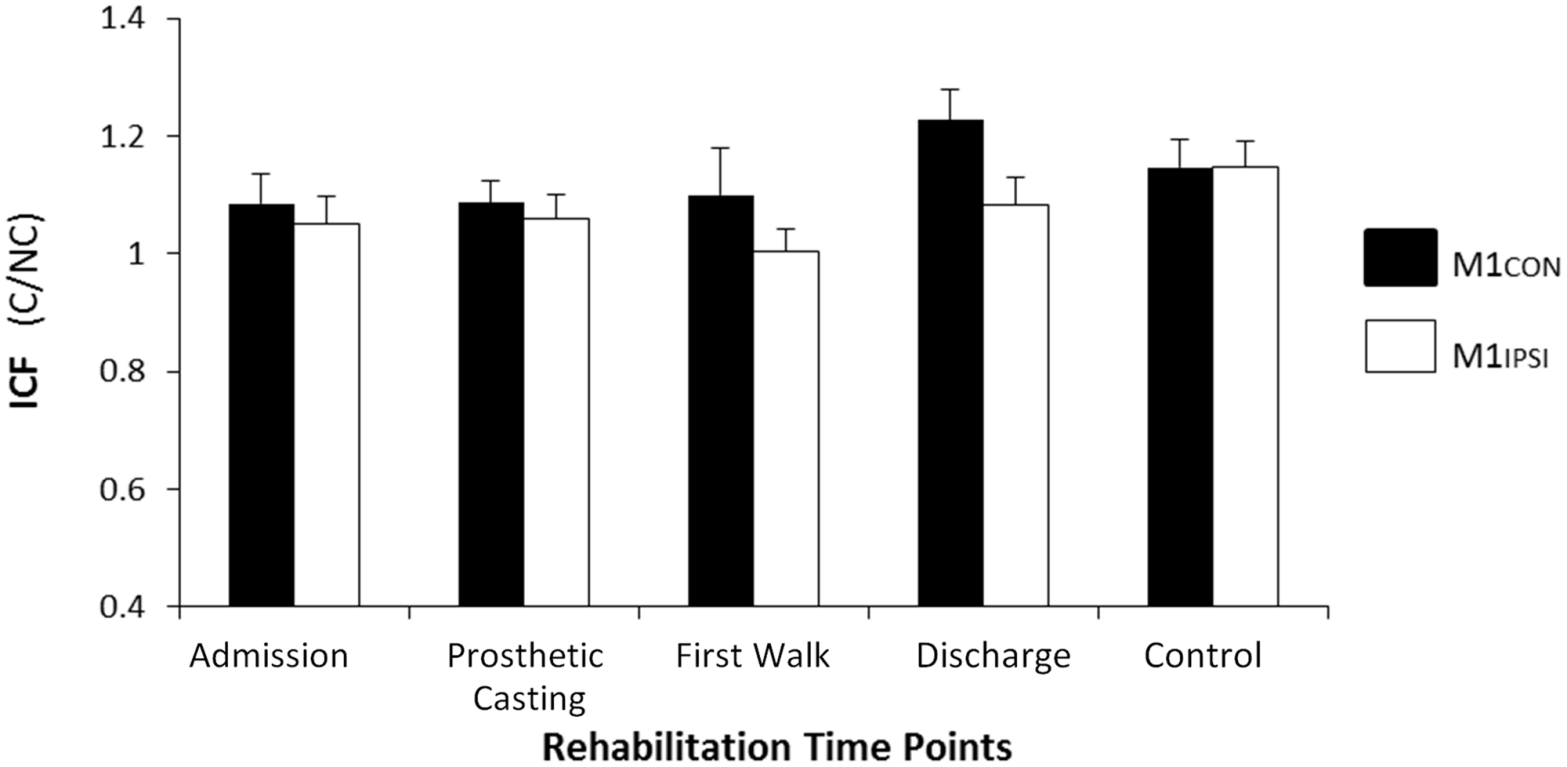
**Modulation of ICF across rehabilitation time points for M1CON and M1IPSI**. There was no significant modulation of ICF across rehabilitation or compared to control participants. Error bars represent standard error. C, conditioned motor evoked potentials; NC, non-conditioned motor evoked potentials.

The LI did not change across rehabilitation (*p* = 0.48; see **Figure 1**). AMT did not vary significantly across session for M1CON (*p* = 0.85) or M1IPSI (*p* = 0.39). For M1CON, there was significant modulation of SICI [*F*_(3,36)_ = 2.47, *p* = 0.05; see **Figure 2**], but not LICI (*p* = 0.58; see **Figure 3**) or ICF (*p* = 0.25; see **Figure 4**). *Post hoc* analysis for SICI found there was disinhibition at first walk compared to discharge [*t*_(12)_ = 3.66, *p* = 0.003]. For M1IPSI, there was significant modulation of SICI [*F*_(3,36)_ = 3.42, *p* = 0.03; see **Figure 2**], and LICI [*F*_(3,36)_ = 2.19, *p* = 0.05; see **Figure 3**], but not ICF (*p* = 0.63; see **Figure 4**). *Post hoc* analysis for SICI found there was disinhibition at admission [*t*_(12)_ = 3.36, *p* = 0.006] and prosthetic casting [*t*_(12)_ = 3.38, *p* = 0.005] compared to discharge. *Post hoc* analysis for LICI found there was disinhibition at admission [*t*_(12)_ = 2.14, *p* = 0.05] and prosthetic casting [*t*_(12)_ = 2.92, *p* = 0.02] compared to first walk. A summary of neurophysiological data is provided in **Table 1**.

**Table 1:** A summary of neurophysiological data across rehabilitation.

	Control	Amputee			
		Admission	Prosthetic Casting	First walk	Discharge
Days post-amputation (median, range)		14 (7–18)	32 (14–41)	50 (32–75)	87 (47–99)
LI (mean, SD)	-0.10 (0.3)	0.04 (0.3)	-0.12 (0.3)	-0.07 (0.4)	0.00 (0.3)
SICI (mean, SD) M1CON	0.86 (0.1)	0.91 (0.1)	0.91 (0.2)	0.96 (0.3)^∗^	0.79 (0.2)^∗^
M1IPSI	**0.89 (0.1)**	**1.03 (0.1)**ˆ	1.03 (0.2)^+^	0.99 (0.3)	0.82 (0.2)ˆ^+^
LICI (mean, SD) M1CON	0.64 (0.3)	0.70 (0.3)	0.66 (0.3)	0.61 (0.4)	0.61 (0.4)
M1IPSI	0.73 (0.3)	0.73 (0.2)^#^	0.76 (0.4)^∼^	0.58 (0.3)^#∼^	0.69 (0.3)
ICF (mean, SD) M1CON	1.15 (0.2)	1.08 (0.2)	1.11 (0.1)	1.12 (0.3)	1.24 (0.2)
M1IPSI	1.14 (0.2)	1.05 (0.2)	1.05 (0.1)	1.00 (0.1)	1.06 (0.2)

*Positive LI values represent greater excitability of M1*CON*, and negative LI values represent greater excitability of M1*IPSI.* SICI, LICI, and ICF are presented as conditioned MEPs normalized to non-conditioned MEPs. Significant differences between amputees and controls are shown in bold. Significant differences across rehabilitation are indicated with symbols. ^∗^Significant difference in SICI in M1*CON* from first walk to discharge. ˆSignificant difference in SICI in M1*IPSI* from admission to discharge. ^+^Significant difference in SICI in M1*IPSI* from cast walk to discharge. ^#^Significant difference in LICI in M1*IPSI* from admission to first walk. ^∼^Significant difference in LICI in M1*IPSI* from cast to first walk. M1*CON*, primary motor cortex contralateral to the amputated limb; M1*IPSI*, primary motor cortex ipsilateral to the amputated limb; LI, laterality index; SICI, short-latency intracortical inhibition; LICI, long-latency intracortical inhibition; ICF, intracortical facilitation*.

There were no significant main effects or interactions for pre-trigger rmsEMG for amputees (all *p* ≥ 0.21) or controls (all *p* ≥ 0.52). For amputees, the average test MEP to assess paired-pulse measures was 0.40 mV (SD 0.35) for M1CON and 0.54 mV (SD 0.39) for M1IPSI. For controls, the average test MEP to assess paired-pulse measures was 0.69 mV (SD 0.32) for M1CON and 0.75 mV (SD 0.75) for M1IPSI. The test MEP amplitude did not vary across sessions for amputees (all *p* ≥ 0.25).

### Association of Cortical Reorganization with Gait Function

**FIGURE 5:**
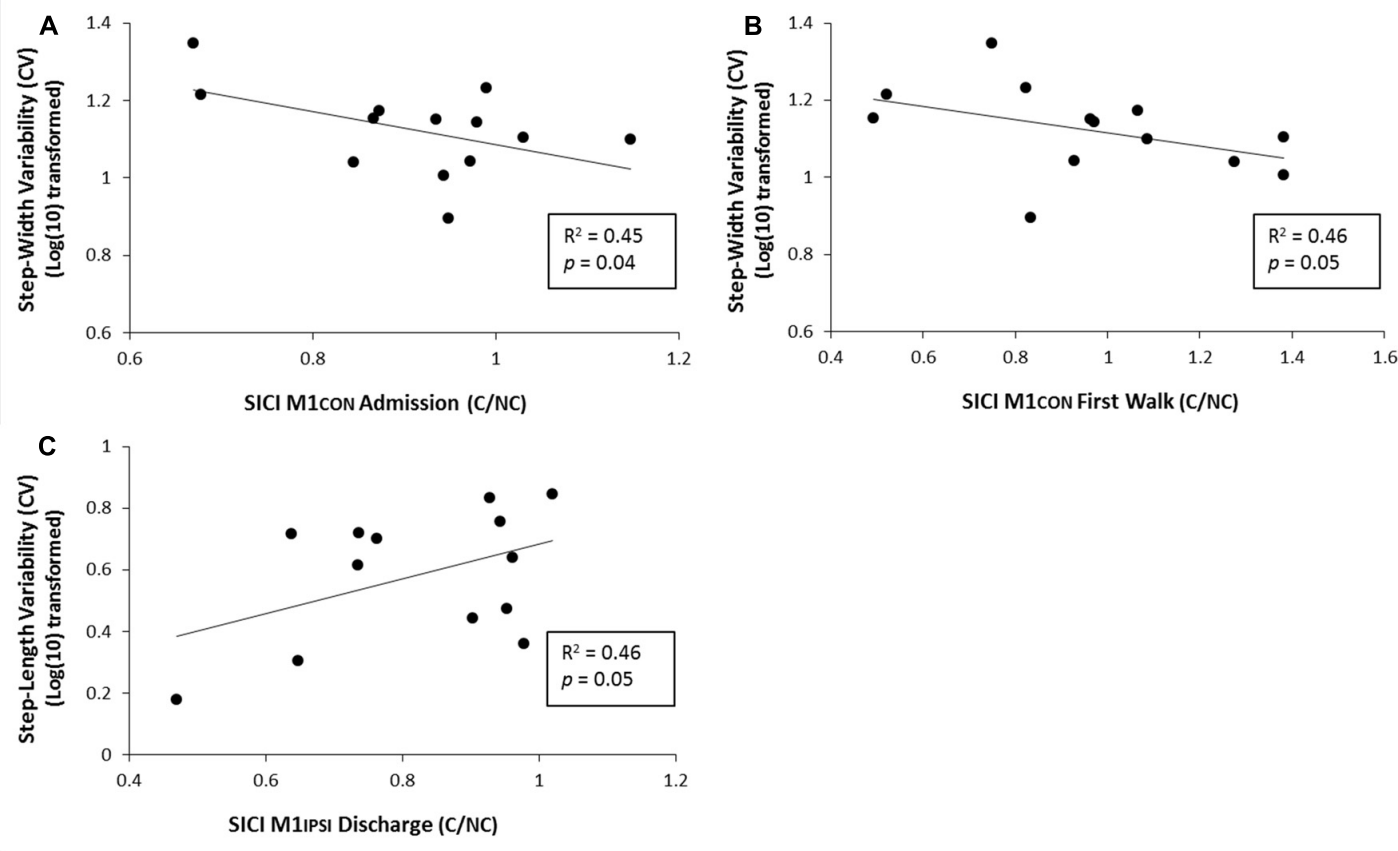
**Linear regression analysis for the relationship between gait variability and SICI at various phases of prosthetic rehabilitation. (A)** Demonstrates a significant negative relationship between step-width variability (log transformed) and SICI in M1CON at admisssion. **(B)** Demonstrates a significant negative relationship between normalized step-width variability (log transformed) and SICI in M1CON at first walk. **(C)** Demonstrates a significant positive relationship between normalized step-length variability (log transformed) and SICI in M1IPSI at discharge.

For amputees, normalized step-length variability was 4.2% (SD 1.8), normalized step-time variability was 9.4% (SD 3.7) and normalized step-width variability was 13.7% (SD 3.6). Linear regression models controlling for age and stump-length found three significant relationships between neurophysiological measures and gait function characterized by gait variability measures. There was a negative relationship between SICI assessed in M1CON at admission and step-width variability at discharge (*R*^2^ = 0.45, *p* = 0.04, unadjusted; see **Figure 5A**). There was a negative relationship between SICI assessed in M1CON at first walk and step-width variability at discharge (*R*^2^ = 0.46, *p* = 0.05, unadjusted; see **Figure 5B**). There was a negative relationship between SICI assessed in M1IPSI at discharge and step-length variability at discharge (*R*^2^ = 0.46, *p* = 0.05, unadjusted; see **Figure 5C**). For all three regression models, no other independent variables controlled for were significant (*p* ≥ 0.10). There were no other significant associations between neurophysiological measures and gait variability measures (all *p ≥* 0.11).

## Discussion

The main findings of this study were that there was longitudinal modulation of intracortical inhibition in M1 bilaterally during prosthetic rehabilitation, which was associated with gait function in this group of transtibial amputees. SICI was reduced in M1CON at first walk and was restored by discharge. SICI and LICI were both reduced in M1IPSI at admission and prosthetic casting. Disinhibition of M1CON at admission and first walk was associated with better gait function at discharge, while disinhibition of M1IPSI at discharge was associated with poorer gait function at discharge. These findings contribute to the understanding of human amputee neurophysiology and may be relevant for future studies seeking to improve rehabilitation outcomes.

At admission to rehabilitation, SICI in M1CON for amputees was similar to controls. However there was significant disinhibition at time of first walk, which was restored by discharge. Pharmacology studies indicate SICI reflects GABA_A_ receptor mediated inhibition, as the administration of GABA_A_ receptor agonists lorazepam (Ziemann et al., 1996; Di Lazzaro et al., 2005) and diazepam (Di Lazzaro et al., 2005) increase SICI. Importantly, the reduction of GABA_A_ receptor mediated inhibition in amputees occurred at the time of learning prosthetic mobility (first walk). It is unlikely that reduced inhibition is related to the loss of afferent input from the amputation as it was not observed at admission, but at first walk, 50 days post-amputation. We suggest the reduction in inhibition is most likely a neuroplastic response partly driven by the process of prosthetic rehabilitation. Reduced GABAergic inhibition is thought to promote conditions conducive to facilitating the induction of synaptic plasticity (Wigstrom and Gustafsson, 1983; Davies et al., 1991; Paulsen and Moser, 1998) and is associated with motor learning (Perez et al., 2004; Stagg et al., 2011). Although motor learning was not assessed in the current study, prosthetic mobility requires motor skill learning and the reduction in SICI may represent enhanced cortical reorganization in response to intense use of the amputated limb during this period. It is likely that the period of rehabilitation from first walk to discharge when the amputee is learning to mobilize with a prosthesis represents a period of significant motor learning. Our results indicate a reduction in intracortical inhibition at this time, suggesting that the cortical environment may be optimal for reorganization. With evidence of a reduction in intracortical inhibition, we propose that the period of learning to mobilize with a prosthesis may be the most important period to maximize functional gains from prosthetic rehabilitation.

Interestingly modulation of intracortical excitability following amputation was not limited to M1CON. There was an even greater reduction in SICI and a reduction in LICI in M1IPSI following amputation across the early phases of rehabilitation. Similar to the contralateral M1, the reduction in SICI indicates GABA_A_ receptor mediated inhibition likely underpins cortical reorganization in M1IPSI. Furthermore, we observed a reduction in LICI at admission and prosthetic casting. Cortical interneurons responsible for LICI directly inhibit corticospinal output via post-synaptic GABA_B_ receptors *and* pre-synaptic GABA_B_ receptors on inhibitory interneurons responsible for SICI (Werhahn et al., 1999; McDonnell et al., 2006). In the current study, both LICI and SICI were decreased in M1IPSI at admission and prosthetic casting and this concurrent reduction likely reflects that LICI was attenuated in the early phase of rehabilitation via reduced post-synaptic GABA_B_ receptor activity. However, at time of first walk we observed reduced SICI and increased LICI in M1IPSI, reflecting increased excitability of pre-synaptic GABA_B_ receptors leading to auto-inhibition.

The finding of reduced GABA_B_ receptor activity in post-acute amputees is consistent with a previous lower limb amputee case study which also reported a reduction in GABA_B_ receptor activity over the entire rehabilitation period in M1IPSI (Hordacre and Bradnam, 2013). However, current results in this larger study suggest a reduction in GABA_B_ receptor activity is more evident in the early phase of rehabilitation as opposed to the later rehabilitation period. Reduced GABA_A_ and GABA_B_ receptor activity in M1IPSI provide additional evidence of bilateral cortical reorganization following unilateral amputation (Capaday et al., 2000; Schwenkreis et al., 2003; Hordacre et al., 2014b). Our findings add to this evidence by demonstrating rehabilitation phase-dependent modulation of GABA_A_ and GABA_B_ receptor activity in the ipsilateral M1. The specific timing of modulation of intracortical inhibition also suggests the critical period for reorganization within M1IPSI is early in the rehabilitation phase prior to receiving an interim prosthesis. Further attention to this pre-prosthetic rehabilitation period may improve rehabilitation outcomes and should be further investigated.

Interestingly our results indicate that the ipsilateral M1 reorganizes earlier than the contralateral M1 and this may reflect functional tasks performed in rehabilitation. The underlying mechanisms leading to the reduction in inhibition of M1 ipsilateral to the amputated limb are difficult to determine from this study. Previous studies indicate lower limb motor control relies on the bilateral cortical contribution from the primary motor cortices (Luft et al., 2002; Sahyoun et al., 2004; Hordacre et al., 2014b). We suggest disinhibition of M1IPSI may reflect a cortical contribution to driving the residual muscles of the amputated lower limb via up-regulation of ipsilateral pathways to the spinal cord. A second, and equally probable explanation, is that disinhibition of M1IPSI represents a use-dependent cortical response to greater reliance by the amputee on the non-amputated limb during their rehabilitation. Use-dependent plasticity may occur prior to provision of a prosthesis due to increased dependence on the non-amputated leg. This hypothesis may explain the earlier reorganization observed for the ipsilateral M1 compared to the contralateral M1. We propose that it is unlikely interhemispheric projections contributed to the reorganization of the ipsilateral M1 observed here. This is supported by the observation that the ipsilateral M1 appears to reorganize earlier than the contralateral M1, and furthermore, our previous work in long-term lower limb amputees (Hordacre et al., 2014b) suggests interhemispheric inhibition is not modulated following amputation.

An interesting outcome of this study was the association between neurophysiology and gait function. The relationship between cortical neurophysiology and gait function at discharge indicate reduced GABA_A_ receptor mediated inhibition within contralateral M1 at admission and first walk are adaptive neurophysiological responses, as they were associated with reduced gait variability. Therefore, reduced GABAergic inhibition at admission and first walk may be an appropriate neurophysiological biomarker of function at discharge. Conversely, an ongoing reduction in GABA_A_ receptor mediated inhibition of the ipsilateral M1 may be a maladaptive neurophysiological response as it was associated with increased gait variability at discharge. Our results indicate normal levels of SICI within ipsilateral M1 should be restored following prosthetic casting and prior to prosthetic mobility to drive optimal recovery of function. Ongoing reorganization of M1IPSI at discharge may interfere with the normal pattern of bilateral cortical motor control of the amputated limb leading to degraded gait function as we demonstrated previously in long-term amputees (Hordacre et al., 2014b). Interventions that address up regulation of descending ipsilateral projections from M1IPSI, which potentially impair control of prosthetic gait, may assist prosthetic rehabilitation.

There are limitations to this study which should be acknowledged. First, it is difficult to evoke MEPs of reasonable amplitude from stimulation of the lower limb motor cortex given the location of the representations relative to the surface of the head. To address this we pre-activated the RF to a standardized and controlled level of activity while performing neurophysiological assessments. Although there was no significant difference in pre-trigger rmsEMG across sessions, muscle strength may have increased over rehabilitation which may have had a minor influence over the neurophysiological assessments. Second, relatively low levels of SICI were observed in this study compared to cortical representations in other muscles, particularly those of the upper-limb. Differences observed between muscle representations are likely the result of functional specificity (Chen et al., 1998b) and the fact we had to pre-activate the RF muscle for TMS. Muscle activation causes a reduction in inhibition (Ridding et al., 1995), however, the intracortical inhibition evoked in our study are similar to that previously reported for the same activated muscle in healthy adults (Sidhu et al., 2013). Finally, time effects cannot be excluded as a confounding variable for our amputee neurophysiology. While motor learning is likely to be the most significant factor contributing the modulation of intracortical excitability observed in this study, future studies should attempt to control for time effects by assessing control subjects at similar time points to the key rehabilitation phases.

## Conclusion

We demonstrated bilateral modulation of intracortical inhibition within M1 at specific time points during prosthetic rehabilitation in post-acute transtibial amputees. For the contralateral M1, reduced GABA_A_ receptor mediated inhibition at first walk may indicate mechanisms underlying adaptive cortical reorganization, leading to improved gait function. The period from first walk to discharge represents a critical time where the conditions for cortical reorganization are facilitated in the contralateral M1. For the ipsilateral M1, both GABA_A_ and GABA_B_ receptor mediated inhibition were reduced earlier in rehabilitation at admission and prosthetic casting. This response may reflect bilateral cortical control of the lower limbs or increased use of the non-amputated limb in the early, pre-prosthetic, rehabilitation phase. Poor gait function was observed when reduced intracortical inhibition in the ipsilateral M1 extends to discharge from rehabilitation. Future studies are required to further investigate intracortical inhibition as a biomarker using non-invasive brain stimulation interventions to ‘normalize’ intracortical inhibition and determine if this improves gait function at discharge.

## Conflict of Interest Statement

The authors declare that the research was conducted in the absence of any commercial or financial relationships that could be construed as a potential conflict of interest.
